# Case series about severe corneal abscesses: Epidemiological, clinical and microbiological study (about 37 cases)

**DOI:** 10.1016/j.amsu.2022.103834

**Published:** 2022-05-20

**Authors:** Khidrou Fadhoullahi Oumarou Sambou, Abdoul Salam Youssoufou Souley

**Affiliations:** a Military Hospital of Instruction Mohamed V of Rabat, Morocco; b Ophthalmology Department of the Army Hospital, Niamey, Niger

**Keywords:** Case series, Corneal abscesses, Risk factors, Blindness

## Abstract

**Introduction:**

corneal abscess is an infiltration of the layers of the cornea of infectious origin, it is an ophthalmological emergency, the objective of our study is to determine the epidemiological, microbiological and evolutive characteristics of corneal abscesses.

**Patients and methods:**

Retrospective, descriptive study of 37 patients hospitalized for corneal abscesses between January 2017 and February 2021 at the military hospital of Rabat.

**Results:**

The average age of the patients in our study was 42 years. The median time before the consultation was 6 days. The most frequent risk factor was contact lens wear at 32.43%. Ocular trauma was reported in 13.51% of cases. Initial visual acuity was less than one tenth in all patients. Culture was positive in 67.56% of cases. The most common germ was Staphylococcus aureus with 32.43%, followed by Pseudomonas aeruginosa with 16.21% of cases. The prognosis was relatively good with prompt and adequate management.

**Conclusion:**

A good preliminary analysis of the risk factors, the mode of infection, and the adapted research of the incriminated germs allow a secondary adequate management of the severe corneal abscesses.

## Highlights

• Contact lens wear is by far the most common risk factor.
• Staphylococcus aureus was the most common germ.
• Better prognosis if treated quickly and appropriately.

## 1 Introduction

Corneal abscesses are a serious and frequent condition with serious complications such as endophthalmitis, perforation up to blindness. The aim of our study was to define the epidemiological, microbiological and evolutionary aspects of corneal abscesses.

## 2 Patient and method

We have conducted a retrospective descriptive study of 37 patients hospitalized for corneal abscesses between January 2017 and February 2021 at the Mohamed V military hospital in Rabat. This study only included hospitalized patients, the criteria for hospitalization were: abscess diameter greater than 2 mm, depth greater than 50% of corneal thickness, central location, anterior chamber inflammation, immunosuppression, poor compliance with the treatment. The presence of only one criteria indicated the hospitalization, otherwise he was excluded from the study. For each patient we collected age, sex, history, favouring factors, hospitalization criteria, visual sequelae, microbiological and therapeutic characteristics which were subjected to statistical analysis. The data were treated with Microsoft Excel 2016. This work has been reported in line with the PROCESS 2020 criteria [1].

## 3 Results

The average age was 42 years, ranging from 16 to 77 years. Sex ratio M/F = 1.17 (20 men/17 women).

The median consultation time was 6 days and the average hospital stay was 16 days.

**Fig. 1:**
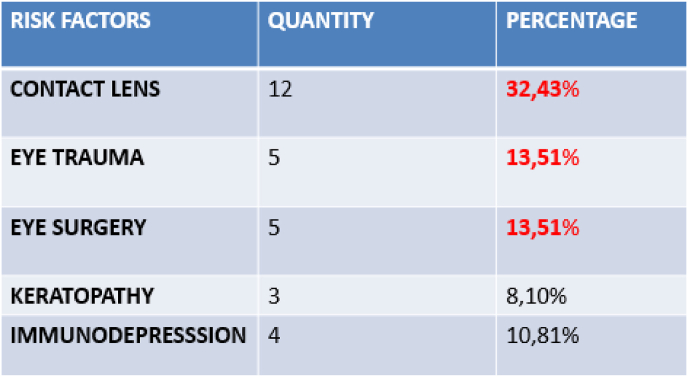
Risk factors found in 78.37%.

A risk factor was identified in 78.37% of the cases with the following frequency: contact lens wearer (32.43%), ocular surgery (13.51%), corneal trauma (13.51%), chronic keratopathy (8.10%), general immunodepression (10.81%) (Fig. 1).

All patients had unilateral damage (left eye = 52.77%; right eye = 47.22%).

Initial visual acuity ranged from light perception to 1/10; the diameter of the abscess was between 2 and 5 mm in 15 patients (40.54%), and greater than 5 mm in 22 cases (59.45%); central location in 28 cases (75.67%), and paracentral location in 9 cases (24.32%); inflammation of the anterior chamber in 70.27%: Tyndall 2x in 13 patients, hypopyon in 10 cases, non-explorable anterior chamber in 3 cases.

**Fig. 2:**
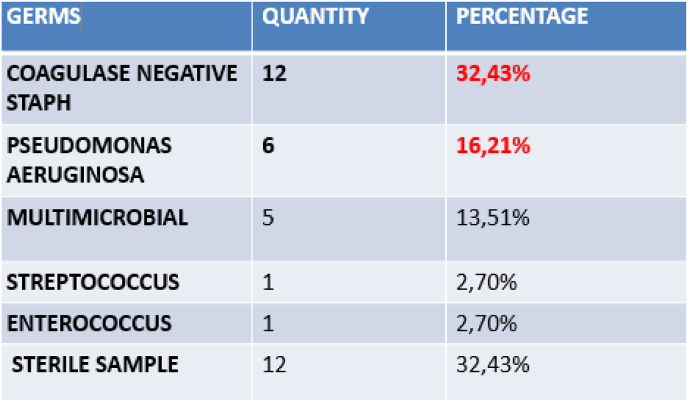
Microbiological aspects: germs identified in 25 cases, i.e. 67.56%.

All patients received a corneal swab and a germ was isolated in 67.56% of cases:coagulase-negative staphylococci (32.43%) and Pseudomonas aeruginosa (16.21%) were the most frequently encountered species, with multi-microbial involvement in 13.88% of cases (Fig. 2).

**Fig. 3:**
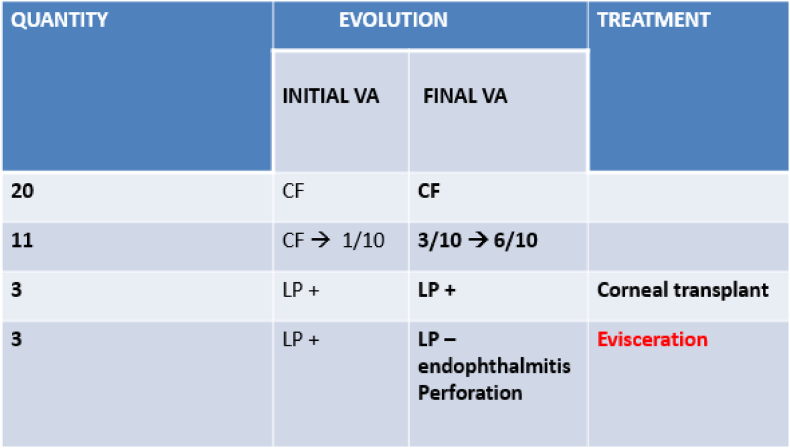
Clinical evolution under treatment. VA: visual acuity; CF:Count fingers; LP: light perception.

All patients received broad-spectrum antibiotic therapy and then adapted to the antibiogram during their hospitalization. The follow-up after 6 months showed the following functional evolution: in 20 patients (54.05%) the initial and final visual acuity remained at counting fingers (CF). In 11 patients (29.72%) there was a good improvement in visual acuity from CF-1/10 to 3/10-6/10. Three patients had a luminous perception even after treatment and received a corneal graft; the remaining 3 patients presented a complication (endophthalmitis and perforation) and had an evisceration performed (Fig. 3).

In our series, complications were noted in 29 cases, i.e. 78.37%. Among the complications, 18 cases of corneal opacity (62.06%), 5 cases of corneal neovascularisation (17.24%), 3 cases of persistent corneal ulcers (10.34%), 2 cases of endophthalmitis, i.e. 6.89% of the complications, and one case of corneal perforation, i.e. 3.44%.

## 4 Discussion

All age ranges were affected with a predilection for young adults (between 20 and 55 years). There was a slight male predominance (sex ratio M/F = 1.17). These results are consistent with the literature on corneal abscesses [2].

The most frequent risk factor was the wearing of contact lenses in 32.43% of the patients in our study. The development of lenses in recent years may explain the increasing incidence of corneal abscesses, with a frequency ranging from 30 to 66% depending on the series [3,4].

**Fig. 4:**
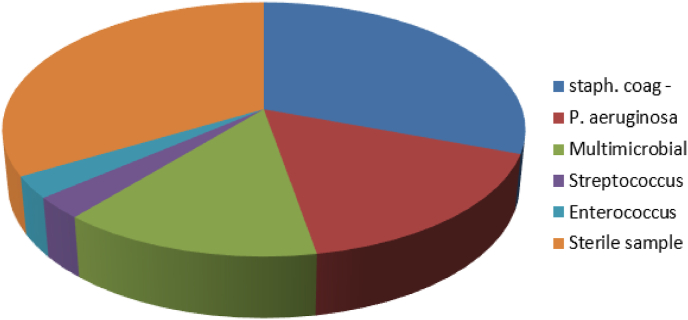
Germ distribution.

**Fig. 5:**
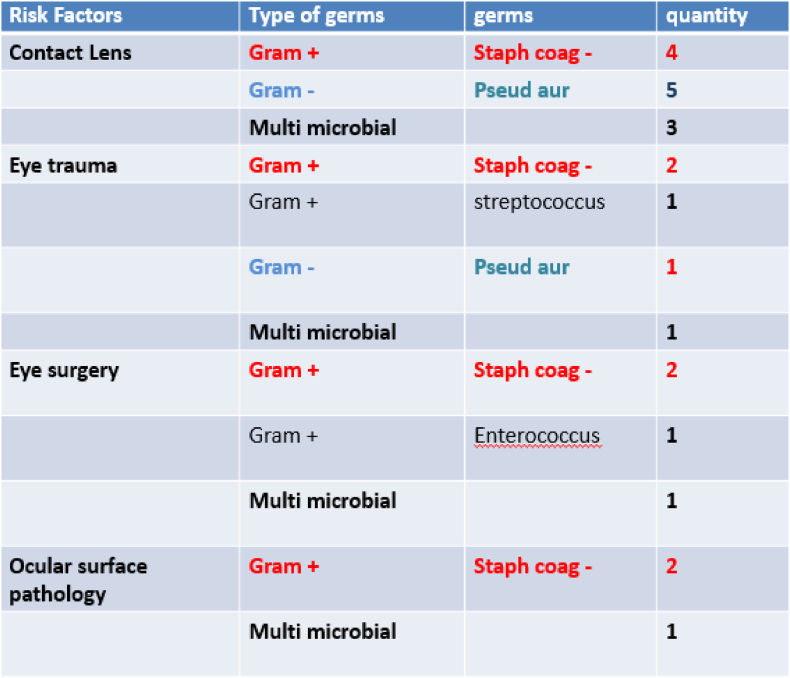
Number of cases of germs found according to risk factors, Note that P.aerurginosa is more frequent in contact lens wearers.

The rate of positivity of corneal cultures varies between 60 and 84% according to the authors [[5], [6], [7]], in our series it is 67.56%, all the germs were bacteria, according to the proportions we distinguish: staphylococcus with negative coagulase in 32.43%, Pseudomonas aeruginosa with 16.21%, streptococcus with 2.7%, enterococcus with 2.7% and multi microbial with 13.51% (Fig. 4). It was also noted that, apart from lens wearers, whatever the other risk factors considered, the most frequent bacteria were gram-positive cocci, in our series it was coagulase-negative staphylococcus (Fig. 5), and these results are in line with those in the literature [8,9].

The sterile samples in these patients are mainly explained by a previous antibiotic therapy that decapitated the infection.

The examination of visual acuity showed that the prognosis was relatively good with an improvement in visual acuity in 29.72% of cases, which is explained by the presence of para-central abscesses and the short consultation time. The contact lens is not a factor of poor prognosis in our series.

Complications were noted in 78.37% of cases, compared to 48.51% in another study [2]. This result can be explained by the delay in consultation but also by the severity of the initial inflammation. Corneal opacity is by far the most frequent complication, altering visual function especially if central abcess.

Patients with immunodepression have all benefited from evisceration.

Among the poor prognostic factors in our study, we note: initial visual acuity, intraocular inflammation, diagnostic delay, immunodepression.

## 5 Conclusion

Corneal abscess is a serious and frequent pathology that can lead to blindness. Only an early management adapted to the microbiological results can improve the prognosis of corneal abscess. The adverse evolution was statistically determined by the extent of the inflammatory reaction in the anterior chamber, the initial visual acuity and immunosuppression.

## Ethical approval

Not related.

## Sources of funding

Not available.

## Author contributions

Corresponding author: OUMAROU SAMBOU KHIDROU FADHLOULLAHI

YOUSSOUFOU SOULEY ABDOUL SALAM. data analysis, study concept.

## Registration of research studies

Not applicable here, this is a study of cases.

## Guarantor

Oumarou Sambou Khidrou Fadhloullahi.

Youssoufou Souley Abdoul Salam.

## Consent

Written informed consent was obtained from the patients for this publication of this study. A copy of the written consent is available for review by the Editor-in-Chief of this journal on request”.

## Provenance and peer review

Not commissioned, externally peer-reviewed.

## Declaration of competing interest

THERE IS NOT CONFLICTS OF INTEREST.

## References

[1] Agha R.A., Sohrabi C., Mathew G., Franchi T., Kerwan A., O'Neill N for the PROCESS Group (2020). The PROCESS 2020 guideline: updating consensus preferred reporting of CasE series in surgery (PROCESS) guidelines. Int. J. Surg..

[2] Masson E. Severe abscesses of the cornea: about 100 cases. https://www.em-consulte.com/article/113438/les-abces-graves-de-la-cornee.

[3] Bourcier T., Thomas F., Borderie V., Chaumeil C., Laroche L. (2003). Bacterial keratitis: predisposing factors, clinical and microbiological review of 300 cases. Br. J. Ophthalmol..

[4] Buehler P.O., Schein O.D., Stamler J.F., Verier D.D., Katz J. (1992). The increased risk of ulcerative keratitis among disposable soft contact lens users. Arch. Ophthalmol..

[5] Clinch T.E., Palmon F.E., Robinson M.J., Cohen E.J., Barron B.A., Laibson P.R. (1994). Microbial keratitis in children. Am. J. Ophthalmol..

[6] Cheung J., Slomovic A.R. (1995). Microbial etiology and predisposing factors among patients hospitalized to corneal ulceration. Can. J. Ophthalmol..

[7] Rodman R.C., Spisak S., Sugar A., Meyer R.F., Kaz Soong L., Musch D.C. (1997). The utility of culturing corneal ulers in a tertiary referral center versus a general ophthalmology clinic. Ophthalmology.

[8] Charteris D.G., Batterbury M., Armstrong M., Tullo A.B. (1994). Suppurative keratitis caused by streptococcus pneumoniae after cataract surgery. Br. J. Ophthalmol..

[9] Cameron J.A., Huaman A. (1994). Corneoscleral abscess resulting from a broken suture after cataract surgery. J. Cataract Refract. Surg..

